# Deep Learning for Plant Identification in Natural Environment

**DOI:** 10.1155/2017/7361042

**Published:** 2017-05-22

**Authors:** Yu Sun, Yuan Liu, Guan Wang, Haiyan Zhang

**Affiliations:** School of Information Science and Technology, Beijing Forestry University, Beijing 100083, China

## Abstract

Plant image identification has become an interdisciplinary focus in both botanical taxonomy and computer vision. The first plant image dataset collected by mobile phone in natural scene is presented, which contains 10,000 images of 100 ornamental plant species in Beijing Forestry University campus. A 26-layer deep learning model consisting of 8 residual building blocks is designed for large-scale plant classification in natural environment. The proposed model achieves a recognition rate of 91.78% on the BJFU100 dataset, demonstrating that deep learning is a promising technology for smart forestry.

## 1. Introduction

Automatic plant image identification is the most promising solution towards bridging the botanical taxonomic gap, which receives considerable attention in both botany and computer community. As the machine learning technology advances, sophisticated models have been proposed for automatic plant identification. With the popularity of smartphones and the emergence of Pl@ntNet mobile apps [1], millions of plant photos have been acquired. Mobile-based automatic plant identification is essential to real-world social-based ecological surveillance [2], invasive exotic plant monitor [3], ecological science popularization, and so on. Improving the performance of mobile-based plant identification models attracts increased attention from scholars and engineers.

Nowadays, many efforts have been conducted in extracting local characteristics of leaf, flower, or fruit. Most researchers use variations on leaf characteristic as a comparative tool for studying plants, and some leaf datasets including Swedish leaf dataset, Flavia dataset, and ICL dataset are standard benchmark. In [4], Söderkvist extracted shape characteristics and moment features of the leaves and analyzed the 15 different Swedish tree classes using back propagation for the feed-forward neural network. In [5], Fu et al. chose the local contrast and other parameters to describe the characteristics of the surrounding pixels of veins. The artificial neural network was used to segment the veins and other leaves. The experiment shows that the neural network is more effective in identifying the vein images. Li et al. [6] proposed an efficient leaf vein extraction method by combining snakes technique with cellular neural networks, which obtained satisfactory results on leaf segmentation. He and Huang used the probabilistic neural network as a classifier to identify the plant leaf images, which has a better identification accuracy comparing to BP neural network [7]. In 2013, the idea of natural-based leaf recognition was proposed, and the method of contour segmentation algorithm based on polygon leaf model was used to obtain contour image [8]. With the deep learning becoming a hot spot in the field of image recognition, Liu and Kan proposed texture features in combination with shape characteristics, using deep belief network architecture as a classifier [9]. Zhang et al. designed a deep learning system which includes eight layers of Convolution Neural Network to identify leaf images and achieved a higher recognition rate. Some researchers focus on the flowers. Nilsback and Zisserman proposed a method of bag of visual word to describe the color, shape, texture features, and other characteristics [10]. In [11], Zhang et al. combined Harr features with SIFT features of flower image, coding them with nonnegative sparse coding method and classifying them by* k*-nearest neighbor method. In [12], they raised a method of recognizing the picking rose by integrating BP neural network. The studies of identifying plants by fruit are relatively rare. Li et al. proposed the method of multifeature integration using preference Ainet as the recognition algorithm [13]. After so many years continued exploration into plant recognition technology, the dedicated mobile applications such as LeafSnap [14], Pl@ntNet [1], or Microsoft Garage's Flower Recognition app [15] can be conveniently used for identify plants.

Although the research on automatic plant taxonomy has yield fruitful results, one must note that those models are still far from the requirements of a fully automated ecological surveillance scenario [3]. The aforesaid datasets lack the mobile-based plant images acquired in natural scene which vary greatly in contributors, cameras, areas, periods of the year, individual plants, and so on. The traditional classification models rely heavily on preprocessing to eliminate complex background and enhance desiring features. What is more, the handcraft feature engineering is incapable of dealing with large-scale datasets consisting of unconstrained images.

To overcome aforementioned challenges and inspired by the deep learning breakthrough in image recognition, we acquired the BJFU100 dataset by mobile phone in natural environment. The proposed dataset contains 10,000 images of 100 ornamental plant species in Beijing Forestry University campus. A 26-layer deep learning model consisting of 8 residual building blocks is designed for uncontrolled plant identification. The proposed model achieves a recognition rate of 91.78% on the BJFU100 dataset.

## 2. Proposed BJFU100 Dataset and Deep Learning Model

Deep learning architectures are formed by multiple linear and nonlinear transformations of input data, with the goal of yielding more abstract and discriminative representations [16]. These methods have dramatically improved the state-of-the-art in speech recognition, visual object recognition, object detection, and many other domains such as drug discovery and genomics [17]. The deep convolutional neural networks proposed in [18] demonstrated outstanding performance in the large-scale image classification task of ILSVRC-2012 [19]. The model was trained on more than one million images and has achieved a winning top-5 test error rate of 15.3% over 1,000 classes. It almost halved the error rates of the best competing approaches. This success has brought about a revolution in computer vision [17]. Recent progress in the field has advanced the feasibility of deep learning applications to solve complex, real-world problems [20].

### 2.1. BJFU100 Dataset

The BJFU100 dataset is collected from natural scene by mobile devices. It consists of 100 species of ornamental plants in Beijing Forestry University campus. Each category contains one hundred different photos acquired by smartphone in natural environment. The smartphone is equipped with a prime lens of 28 mm equivalent focal length and a RGB sensor of 3120 × 4208 resolution.

**Figure 1:**
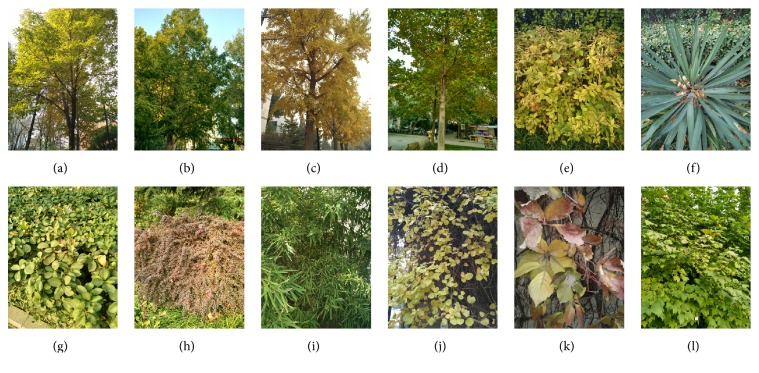
Example images of the BJFU100 dataset. (a) Chinese buckeye, (b) metasequoia, (c)* Ginkgo biloba*, (d) hybrid tulip tree, (e)* Weigela florida* cv. red-prince, (f)* Yucca gloriosa,* (g)* Euonymus kiautschovicus* Loes, (h)* Berberis thunbergii* var. atropurpurea, (i) mottled bamboo, (j)* Celastrus orbiculatus,* (k)* Parthenocissus quinquefolia, *and (l)* Viburnum opulus*.

For tall arbors, images were taken from a low angle at ground as shown in Figures 1(a)–1(d). Low shrubs were shot from a high angle, as shown in Figures 1(e)–1(h). Other ornamental plants were taken from a level angle. Subjects may vary in size by an order of magnitude (i.e., some images show only the leaf, others an entire plant from a distance), as shown in Figures 1(i)–1(l).

### 2.2. The Deep Residual Network

With the network depth increasing, traditional methods are not as expected to improve accuracy but introduce problems like vanishing gradient and degradation. The residual network, that is, ResNet, introduces skip connections that allow the information (from the input or those learned in earlier layers) to flow more into the deeper layers [23, 24]. With increasing depth, ResNets give better function approximation capabilities as they gain more parameters and successfully contribute to solving vanishing gradient and degradation problems. Deep residual networks with residual units have shown compelling accuracy and nice convergence behaviors on several large-scale image recognition tasks, such as ImageNet [23] and MS COCO [25] competitions.

#### 2.2.1. Residual Building Blocks

**Figure 2:**
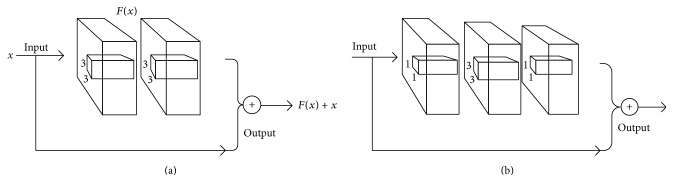
(a) A basic building block. (b) A “bottleneck” building block of deep residual networks.

Residual structural unit utilizes shortcut connections with the help of identity mapping. Shortcut connections are those skipping one or more layers. The original underlying mapping can be realized by feed-forward neural networks with shortcut connections. The building block illustrated in Figure 2 is defined as

(1) y=Fx,Wi+x,F=W2σW1x,σa=max⁡0,a,

where *x* and *y* are the input and output vectors of stacked layers, respectively. The function *F*(*x*, {*W*_*i*_}) represents the residual mapping that needs to be learned. The function *σ*(*a*) denotes ReLU [26] and the biases are omitted for simplifying notations. The dimensions of *x* and *F* must be equal to perform the element-wise addition. If this is not the case, a linear projection *W*_*s*_ is applied to match the dimensions of *x* and *F*:

(2) $$\begin{array}{c} y=F\left(\;x,\left\{\;W_{i}\;\right\}\;\right)+W_{s}x. \end{array}$$

The baseline building block is shown in Figure 2(a). A shortcut connection is added to each pair of 3 × 3 filters. Concerning the training time on deeper nets, a bottleneck building block is designed as in Figure 2(b). The three layers are 1 × 1, 3 × 3, and 1 × 1 convolutions, where the 1 × 1 layers are responsible for reducing and then restoring dimensions, leaving 3 × 3 layer a bottleneck with smaller input/output dimensions [23]. Bottleneck building blocks use fewer parameters to obtain more abstraction of layers.

**Figure 3:**
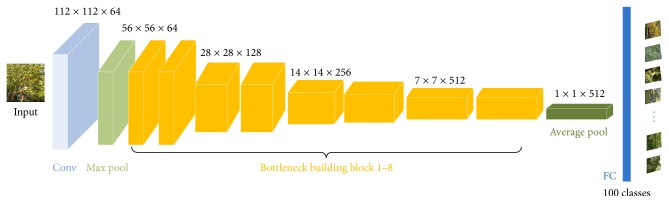
Architecture of 26-layer ResNet model for plant identification.

The overall network architecture of our 26-layer ResNet, that is, ResNet26, model is depicted in Figure 3. As Figure 3 shows, the model is mainly designed by using bottleneck building blocks. The input image is fed into a 7 × 7 convolution layer and a 3 × 3 max pooling layer followed by 8 bottleneck building blocks. When the dimensions increase, 1 × 1 convolution is used in bottleneck to match dimensions. The 1 × 1 convolution enriches the level of abstraction and reduces the time complexity. The network ends with a global average pooling, a fully connected layer, and a softmax layer. We adopt batch normalization (BN) [27] right after each convolution layer and before ReLU [26] activation layer. Downsampling is performed by the first convolution layer, the max pooling layer, and the 3, 5, and 7 bottleneck building blocks.

## 3. Experiments and Results

### 3.1. Implementation and Preprocess

The model implementation is based on the open source deep learning framework keras [28]. All the experiments were conducted on a Ubuntu 16.04 Linux server with a 3.40 GHz i7-3770 CPU (16 GB memory) and a GTX 1070 GPU (8 GB memory). The 100 samples of each class are split into 80 training samples and 20 test samples. Compared with conventional classification methods, data preprocess on deep learning approaches is much simpler. In this paper, the inputs to the network are RGB color images. All the images only need to be rescaled to 224 × 224 pixels and then per-pixel value is divided by 255.

### 3.2. Training Algorithm

During the back propagation phase, the model parameter is trained by the stochastic gradient descent (SGD) algorithm, with the categorical cross-entropy loss function as optimization object. The SGD can be expressed as follows:

(3) δx=wx+1σ′wx+1•cx+bx+1∘upδx+1,Δwx=−η•∑i,jδx∘downSx−1,

where *δ*_*x*_ is sensitivity, *w*_*x*+1_ is multiplicative bias, ∘ indicates that each element is multiplied, up is upsampling, down is downsampling, Δ*w*_*x*_ represents the weight update of the layer, and *η* is the learning rate. The cross-entropy loss function is defined to be

(4) $$\begin{array}{c} L_{i}=-\log\left(\;\frac{e^{f_{y_{i}}}}{\sum_{j}e^{f_{j}}}\;\right), \end{array}$$

where *f*_*j*_ is the *j*th element in the classification score vector *f*.

**Figure 4:**
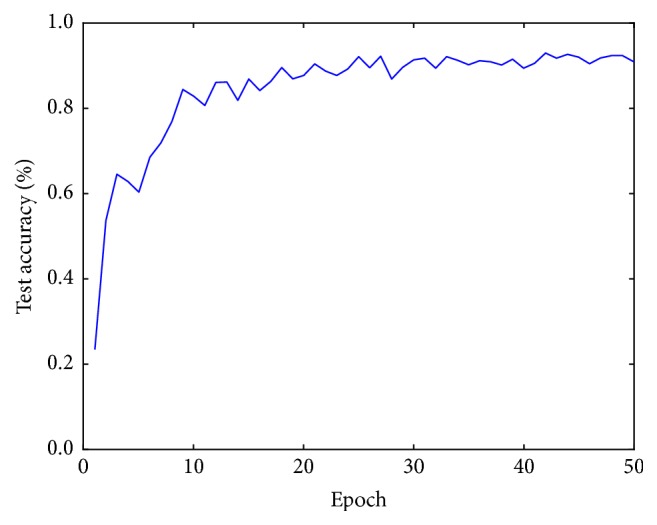
Evolution of classification accuracy in the test set.

After some preliminary training experiments, the base learning rate is set to 0.001, which is gradually reduced at each epoch. The decay rate is 10^−6^ and the momentum is 0.9.  Figure 4 shows the training process of ResNet26 model. Test accuracy improves quickly since the first epochs and stabilizes after 40 epochs.

### 3.3. Results Analysis

**Figure 5:**
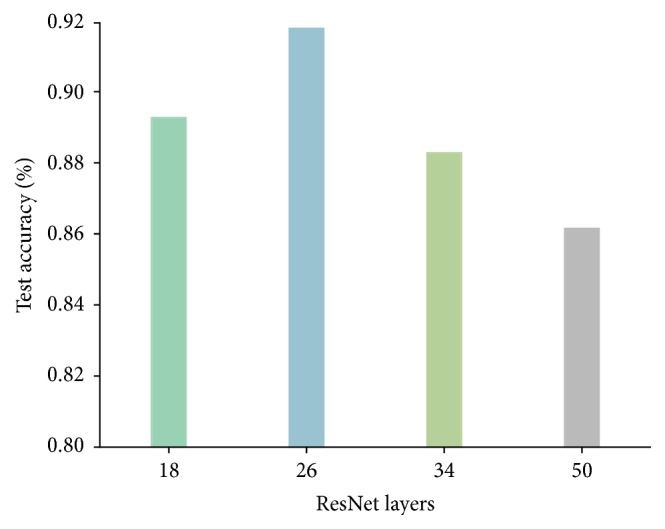
Test accuracy of the ResNet18, ResNet34, ResNet50 [23], and ResNet26 model. The proposed ResNet26 outperforms the best reference ResNet by 2.51%.

To find the best deep residual network, a series of experiments have been conducted on BJFU100 dataset.  Figure 5 shows the comparison of test accuracy among the proposed ResNet26 model and the original ResNet model of 18, 34, and 50 layers [23] designed for ImageNet. The ResNet18, ResNet34, and ResNet50 yield a test accuracy of 89.27%, 88.28%, and 86.15%, respectively. The proposed ResNet26 results in 91.78% accuracy which increases the overall efficiency up to 2.51%.

The ResNet26 is the best tradeoff between model capacity and optimization difficulty. For the size of BJFU100, ResNet26 contains enough trainable parameter to learn the discriminative feathers, which prevents underfitting. Compared to larger model, ResNet26 results in fast and robust convergence during SGD optimization, which prevents overfitting or falls into local optimum.

## 4. ResNet26 on Flavia Dataset

**Figure 6:**
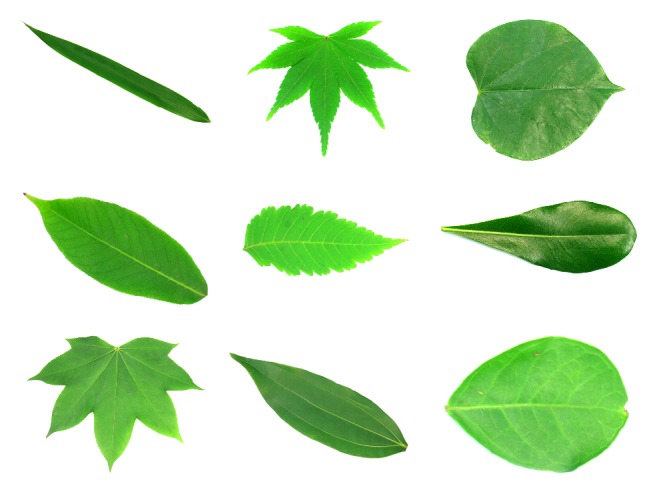
Example images of the Flavia dataset.

To show the effectiveness of the proposed ResNet26 model, a series of experiments have been performed on the publicly available Flavia [29] leaf dataset. It comprises 1907 images of 1600 × 1200 pixels, with 32 categories. Some of the samples are shown in Figure 6. We randomly select 80% of the dataset for training and 20% for testing.

All the images are doubled and resized to 224 × 224 pixels. Per-pixel value is divided by the maximum value and subtracted the mean values of the data.

**Figure 7:**
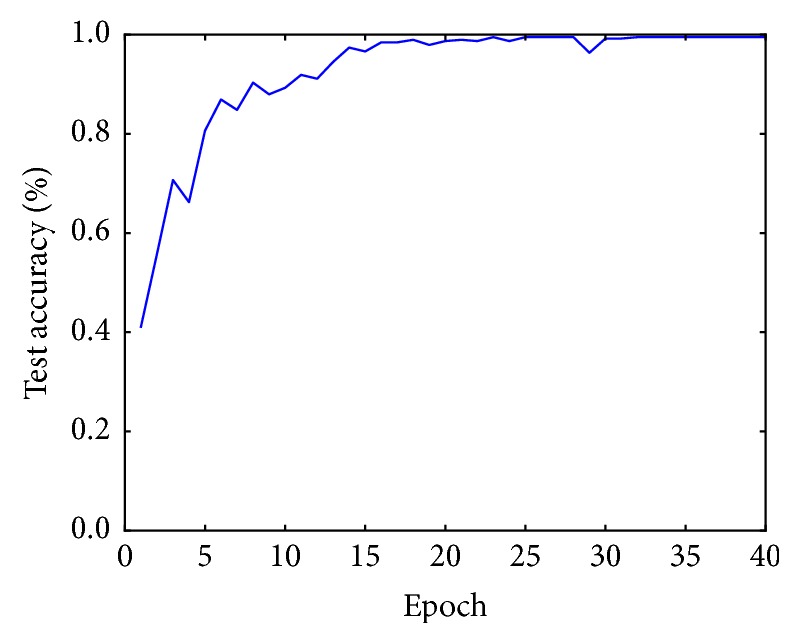
Evolution of classification accuracy in the test set.

The training algorithm is exactly the same as that applied to the BJFU100 dataset.  Figure 7 shows the training process of ResNet26 model. Test accuracy improves quickly since the first epochs and stabilizes after 30 epochs.

**Figure 8:**
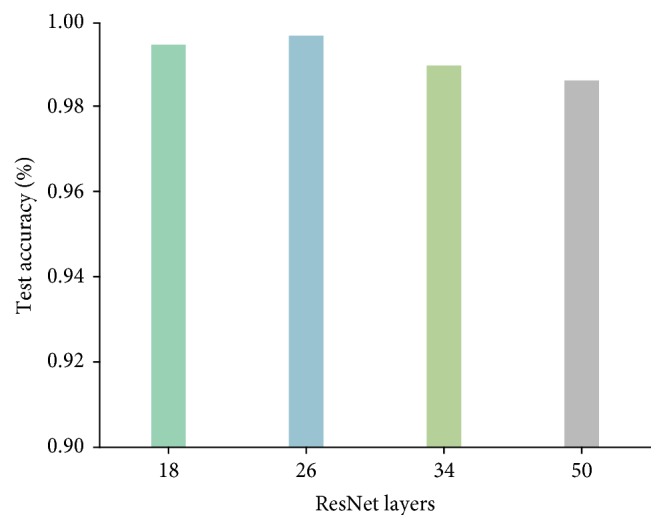
Test accuracy of the ResNet18, ResNet34, ResNet50 [23], and ResNet26 model on Flavia dataset. The proposed ResNet26 outperforms the best reference ResNet by 0.21%.

The test accuracy of each model is estimated by 10-fold cross-validation, as visualized in Figure 8. The ResNet18, ResNet34, and ResNet50 achieve a test accuracy of 99.44%, 98.95%, and 98.60%, respectively. The proposed ResNet26 gains 99.65% accuracy which increases the overall efficiency up to 0.21%.  Table 1 summarizes our result and other previously published results on Flavia [29] leaf dataset. The ResNet26 model achieves a 0.28% improvement compared with the best-performing method.

## 5. Conclusion

The first mobile device acquired BJFU100 dataset containing 10,000 images of 100 plant species which provides data pillar stone for further plant identification study. We continue to expand the BJFU100 dataset by wider coverage of species and seasons. The dataset is open for academic community, which is available at http://pan.baidu.com/s/1jILsypS. This work also studied a deep learning approach to automatically discover the representations needed for classification, allowing use of a unified end-to-end pipeline for recognizing plants in natural environment. The proposed model ResNet26 results in 91.78% accuracy in test set, demonstrating that deep learning is the promising technology for large-scale plant classification in natural environment.

In future work, the BJFU100 database will be expanded by more plant species at different phases of life cycle and more detailed annotations. The deep learning model will be extended from classification task to yield prediction, insect detection, disease segmentation, and so on.

## Figures and Tables

**Table 1:** Recognition rate comparison on Flavia dataset.

Method	Recognition rate
PBPNN [21]	93.82%
SVM [22]	96.00%
DBNs (with “dropout”) [9]	99.37%
Our work	99.65%

## References

[1] Joly A., Goëau H., Bonnet P. (2014). Interactive plant identification based on social image data. *Ecological Informatics*.

[2] Goëau H., Bonnet P., Joly A. LifeCLEF plant identification task 2015.

[3] Goëau H., Bonnet P., Joly A. Plant identification in an open-world (lifeclef 2016).

[4] Söderkvist O. (2001). *Computer Vision Classification of Leaves from Swedish Trees*.

[5] Fu H., Chi Z., Chang J., Fu C. (2003). Extraction of leaf vein features based on artificial neural network—Studies on the living plant identification I. *Chinese Bulletin of Botany*.

[6] Li Y., Zhu Q., Cao Y., Wang C. A leaf vein extraction method based on snakes technique.

[7] He P., Huang L. (2008). Feature extraction and recognition of plant leaf. *Journal of Agricultural Mechanization Research*.

[8] Cerutti G., Tougne L., Mille J., Vacavant A., Coquin D. (2013). Understanding leaves in natural images - a model-based approach for tree species identification. *Computer Vision and Image Understanding*.

[9] Liu N., Kan J.-M. (2016). Plant leaf identification based on the multi-feature fusion and deep belief networks method. *Journal of Beijing Forestry University*.

[10] Nilsback M.-E., Zisserman A. (2010). Delving deeper into the whorl of flower segmentation. *Image and Vision Computing*.

[11] Zhang C., Liu J., Liang C., Huang Q., Tian Q. (2013). Image classification using Harr-like transformation of local features with coding residuals. *Signal Processing*.

[12] Wang Y. J., Zhang Y. W., Wang D. L., Yin X., Zeng W. J. (2014). Recognition algorithm of edible rose image based on neural network. *Journal of China Agricultural University*.

[13] Li X., Li L., Gao Z., Zhou J., Min S. (2012). Image recognition of camellia fruit based on preference for aiNET multi-features integration. *Transactions of the Chinese Society of Agricultural Engineering*.

[14] Kumar N., Belhumeur P. N., Biswas A. Leafsnap: a computer vision system for automatic plant species identification.

[15] https://www.microsoft.com/en-us/research/project/flowerreco/

[16] Bengio Y., Courville A., Vincent P. (2013). Representation learning: a review and new perspectives. *IEEE Transactions on Pattern Analysis and Machine Intelligence*.

[17] LeCun Y., Bengio Y., Hinton G. (2015). Deep learning. *Nature*.

[18] Krizhevsky A., Sutskever I., Hinton G. E. (2012). Imagenet classification with deep convolutional neural networks. *Advances in neural information processing systems*.

[19] http://www.image-net.org/challenges/LSVRC/2012/

[20] Huval B., Wang T., Tandon S. An empirical evaluation of deep learning on highway driving. https://arxiv.org/abs/1504.01716.

[21] Kulkarni A., Rai H., Jahagirdar K., Upparamani P. (2013). A leaf recognition technique for plant classification using RBPNN and Zernike moments. *International Journal of Advanced Research in Computer and Communication Engineering*.

[22] Sari C., Akgül C. B., Sankur B. Combination of gross shape features, fourier descriptors and multiscale distance matrix for leaf recognition.

[23] He K., Zhang X., Ren S., Sun J. Deep residual learning for image recognition.

[24] He K., Zhang X., Ren S., Sun J. Identity mappings in deep residual networks.

[25] Dai J., He K., Sun J. Instance-aware semantic segmentation via multi-task network cascades.

[26] Nair V., Hinton G. E. Rectified linear units improve restricted boltzmann machines.

[27] Ioffe S., Szegedy C. Batch normalization: Accelerating deep network training by reducing internal covariate shift. https://arxiv.org/abs/1502.03167.

[28] https://keras.io/

[29] Wu S. G., Bao F. S., Xu E. Y., Wang Y.-X., Chang Y.-F., Xiang Q.-L. A leaf recognition algorithm for plant classification using probabilistic neural network.

